# Etiologies of Mild and Moderate Diarrheal Illness among Children in Consuelo, Dominican Republic

**DOI:** 10.4269/ajtmh.23-0299

**Published:** 2024-01-02

**Authors:** Ingrid Japa, Derartu Ahmed, Anabel Fernandez, Angie Alvarez, Shirley Sabino Japa, Ramona Cordero, Francia Acosta, Andrew P. Steenhoff, Elizabeth D. Lowenthal

**Affiliations:** ^1^ Niños Primeros en Salud, Consuelo, Dominican Republic;; ^2^ Campus Universidad Central del Este, San Pedro de Macorís, Dominican Republic;; ^3^ University of Pennsylvania, Philadelphia, Pennsylvania;; ^4^ Children’s Hospital of Philadelphia Global Health Center, Philadelphia, Pennsylvania

## Abstract

Since the rotavirus vaccine was included in the Dominican Republic’s national immunization schedule in 2012, the microbiologic etiologies of acute gastroenteritis have not been described. This study aimed to determine the contribution of rotavirus as an etiology of acute gastroenteritis over a 12-month period in children under 5 years of age in both an inpatient and an outpatient setting in Consuelo, Dominican Republic. All children who were seen at Niños Primeros en Salud clinic or admitted to Hospital Municipal Dr. Angel Ponce Pinedo for acute gastroenteritis during January 2021–April 2022 were enrolled in the study. Stools were evaluated for rotavirus, enteric parasites, and pathogenic bacteria. Pathogen detection was compared between outpatients and inpatients and on the basis of child’s vaccination status. From 181 children enrolled, 170 stool samples were collected, 28 (16.5%) from inpatients and 142 (83.5%) from outpatients. Rotavirus was the most commonly detected pathogen and was proportionately more common among hospitalized children, with nine (32.1%) cases among hospitalized children and 16 (11.3%) among outpatient children. (Pearson χ^2^ = 8.1, *P* = 0.004). Among patients with a positive rotavirus result, vaccination rate was lower among moderate (hospitalized) (three of six; 50%) compared with mild (outpatient) diarrhea patients (12 of 15; 80%). *Giardia lamblia* (10%) was the next most prevalent pathogen detected in both inpatients and outpatients using standard laboratory measures. Despite the availability of rotavirus vaccination, rotavirus remains a common cause of gastrointestinal illness among children under 5 years of age in our cohort. Incomplete vaccination status was associated with hospitalization for gastrointestinal illness.

## INTRODUCTION

Before the introduction of rotavirus vaccines to the global market, rotavirus was the leading cause of acute gastroenteritis among children globally, causing an estimated 528,000 deaths every year among children under 5 years of age.^1^ To combat rotavirus and acute gastroenteritis-related hospitalization among children, two live attenuated oral vaccines, Rotarix (RV1; GSK Biologics, Rixensart, Belgium) and RotaTeq (RV5: Merck & Co, Kenilworth, NJ) were licensed for use after clinical trial data from the Americas and Europe showed 85% to 98% efficacy and a good safety profile.^2^^,^^3^ Countries in Latin America were among the first to implement routine vaccination against rotavirus.^4^ Subsequently, studies describing trends in deaths, hospitalizations, and healthcare visits due to acute gastroenteritis and rotavirus related hospitalization have been published from Latin American countries and the Caribbean including El Salvador, Bolivia, Brazil, Nicaragua, Haiti, Colombia, Panama, and Mexico.^5^^–^^17^ However, similar studies have not yet been published related to the impact of rotavirus immunization in the Dominican Republic.

Similarly, recent publications related to nonrotavirus causes of acute gastroenteritis in children are not available for the region. However, historical regional data indicate that other viruses, such as norovirus, adenovirus, and astrovirus, as well as bacterial and parasitic infections, such as enterotoxigenic *Escherichia coli*, *Shigella*, *Salmonella*, and *Cryptosporidium* sp. have been commonly implicated as causes of acute gastroenteritis in young children.^14^^,^^15^

In the Dominican Republic, rotavirus vaccine was included in the national immunization program in 2012.^18^ According to WHO reports, national immunization coverage of rotavirus vaccine in 2021 in the Dominican Republic was 80% among 1-year-olds.^19^ However, rates of rotavirus and other common gastroenteritis pathogens and hospitalization in young children after the introduction of rotavirus vaccine are still lacking in the Dominican Republic.^20^ Thus, this study aimed to determine the contribution of rotavirus as an etiology of acute gastroenteritis over a 12-month period in children aged 0 to 5 years in both an inpatient and an outpatient setting in Consuelo, Dominican Republic after rotavirus vaccine introduction. Additionally, the study explores the presence of coinfection and single-pathogen infection in rotavirus-negative and rotavirus-positive cases to determine what other potential pathogens were present.

## MATERIALS AND METHODS

### Study setting and participants.

Between January 2021 and May 2022, children aged 0 through 5 years were enrolled from one outpatient and one inpatient facility in the Eastern Dominican Republic: Niños Primeros en Salud (NPS) clinic and Hospital Municipal Dr. Angel Ponce Pinedo. Both recruitment sites are in Consuelo, a municipality of approximately 60,000 people in San Pedro de Macorís, Dominican Republic. The NPS clinic provides primary pediatric care to children under age 5 years from 11 of the poorest neighborhoods of Consuelo (Filiu, La 41, Enriquillo, Cachipero, Carretera, Kilombo, Puerto Rico, Puerto Principe, Villa Verde, Los Guandules, and La Mina). The NPS clinic was established in 2009 as a collaboration between the Centro de Salud Divina Providencia and the Children’s Hospital of Philadelphia to provide free care to children living in these underserved areas. Hospital Municipal Dr. Angel Ponce Pinedo is a 20-bed primary care hospital that is part of the public health system and serves both NPS patients and others in the region who require hospitalization, including communities around Consuelo such as the more rural batey communities. Bateyes are shanty-town communities where migrant workers, often sugarcane cutters, live. People in the batey communities live in extreme poverty, which often affects their ability to access resources needed for receiving adequate health care.^21^ The initial plan for this study was to collect data for a 12-month period. However, data collection was later extended when additional funds were procured to allow for expansion of the study methodology to include stool polymerase chain reaction (PCR) testing for 70 children with acute diarrhea.

### Data and sample collection.

**Figure 1.**
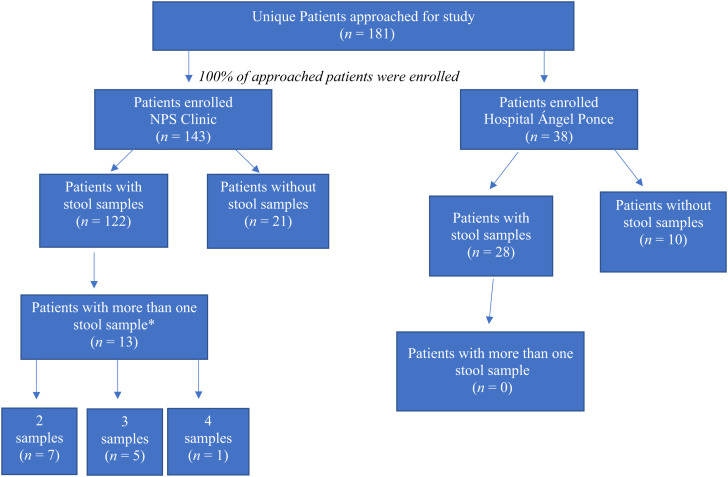
Flow diagram showing participant enrollment and completion (i.e., provision of stool for testing) rates at the Niños Primeros en Salud clinic and Hospital Ángel Ponce. *Some clinic patients presented with more than one acute diarrheal episode during the study. The study team attempted to collect a separate stool sample for each acute diarrheal episode. Thus, 33 samples were provided by 13 unique patients.

The study attempted to enroll all children with acute gastroenteritis who were seen at the NPS clinic or were admitted to the Hospital Ángel Ponce. Acute gastroenteritis was defined as the passage of three or more loose stools per day for less than 14 days, in accordance with WHO guidelines.^22^ To be eligible, children had to be younger than 5 years. Children with more than one episode of acute gastroenteritis during the study period were reassessed by the study team during each illness (Figure 1). All participants were contacted by a study team member upon admission to the hospital or the clinic. If eligible and informed consent was obtained from the caregiver, they were enrolled in the study. The children were enrolled in the study Monday through Friday from 8:00 am to 3:00 pm at both sites. Children were not seen in the clinic at other times. Children could be admitted to the hospital outside of those hours, and all hospital admissions were recorded in a hospital logbook as part of standard of care. The hospital logbook was checked to ensure that all eligible children were approached for possible study inclusion. Children who were admitted after enrollment hours or on the weekends were approached by the study team before discharge from the hospital.

Parents of enrolled children answered questions related to their child’s demographics, health history, symptoms, and vaccinations. Parents were asked to provide a stool sample from their child using globe scientific 30-mL Fecal Tube polypropylene nonsterile (Versatile Scientific Products, Port Jefferson, NY). Stool samples were brought for processing within 1 hour of collection and processed within 5 hours of collection. When the fecal tube arrived in the laboratory, the stool was placed in Carey Blair Transport Medium (Remel Thermo Fisher, Lenexa, KS), and stool samples were processed at accredited laboratories in the Dominican Republic that follow Clinical and Laboratory Standards guidelines for stool bacterial and parasite testing.^23^^,^^24^

### Definitions.

Weight-for-height of less than –2 SD, typically indicative of acute malnutrition, was defined as wasted. Height-for-age of less than –2 SD, commonly indicative of chronic malnutrition, was defined as stunting. Weight-for-height of greater than +2 SD was defined as overweight.

### Laboratory analysis.

Fecal samples were sent to Amadita Laboratorio Clínico or Referencia Laboratorio Clínico, both laboratories accredited by College of American Pathologists. The following tests were conducted in one of the accredited laboratories: occult blood testing using Dencoccult III (Alere, Orlando Florida); rapid testing for rotavirus, *Cryptosporidium species*, *Giardia lamblia*, and *Entamoeba histolytica* using CerTest rapid test (cerTest, Zaragoza Spain); and fecal cultures using Hektoen enteric Agar and MacConkey Sorbitol Agar. Additionally, stool microscopy was used to identify ova and parasitic organisms.

The primary objective of the testing was to determine the prevalence and seasonality of rotavirus infection in both inpatient and outpatient children with acute diarrhea. The secondary objectives were to determine the causes of acute diarrhea in children who did not have rotavirus infection and to determine whether certain pathogens were more common among hospitalized children (i.e., those with moderate to severe illness) than among outpatients (i.e., those with mild symptoms). We also explored whether vaccination rates were lower among inpatients with rotavirus compared with outpatients with rotavirus.

With initial testing, a large proportion of samples failed to reveal any pathogen. Therefore, we sought additional study funding to conduct stool PCR testing to help elucidate which pathogens were being missed by standard testing. The BioFire FilmArray gastrointestinal (GI) panel was then added for the final 70 participants. The number of samples that underwent PCR testing was determined based on available funds. The BioFire GI panel can detect *Campylobacter* (*C. jejuni*/*C. coli*/*C. upsaliensis*), *Clostridioides* (*Clostridium*) *difficile* (toxin A/B), *Plesiomonas shigelloides*, *Salmonella*, *Yersinia enterocolitica*, *Vibrio* (*V. parahaemolyticus/V. vulnificus/V. cholerae*), Enteroaggregative *E. coli*, Enteropathogenic *E. coli*, Enterotoxigenic *E. coli lt/st*, *Shiga-like toxin-producing E. coli stx1/stx2* (*E. coli* O157), *Shigella/*Enteroinvasive *E. coli*, *Cryptosporidium* sp., *Cyclospora cayetanesis*, *Entamoeba histolytica*, *Giardia lamblia*, adenovirus F40/41, astrovirus, norovirus GI/GII, rotavirus A, and sapovirus (I, II, IV, and V).^25^

### Statistical analysis.

Study data was collected using physical form and was later managed using REDCap (Research Electronic Data Capture Tools) hosted at University of Pennsylvania. All analyses were performed using Stata (version 17, StataCorp LLC, College Station, TX). Baseline characteristics were analyzed using descriptive statistics with nonparametric continuous variables reported with medians and interquartile ranges and parametric data reported with means and standard deviations. Normality was assessed using Shapiro–Wilk test. Categorical variables were reported as frequencies (percent). Pearson χ^2^ test was used to evaluate differences between characteristics of enrolled children in the inpatient and outpatient populations. The χ^2^ test (or Fisher’s exact test with frequency < 5) was used to evaluate the association between pathogen detection and healthcare setting.

With > 95% of children in the clinic cohort known to have been vaccinated against rotavirus in recent years, we predicted that ∼10% of clinic outpatients with acute diarrhea would have rotavirus.^26^^,^^27^ We expected to have at least 200 children with diarrhea during the 1-year enrollment period with approximately a 2:1 ratio between outpatients and inpatients. Because unvaccinated children are more likely to have severe rotavirus, we anticipated that the prevalence of rotavirus might be twice as high among inpatients. With the anticipated number of enrollments, we expected to have a 95% CI of no more than 10% on either side of the point estimate for each clinical setting. With a ratio of nonhospitalized to hospitalized children of 2:1, and at least 200 patients enrolled, we expected to have greater than 80% power to detect a relative risk of 1.9 or greater that the inpatients had rotavirus.

## RESULTS

### Participants.

One hundred eighty-one unique patients were enrolled (Figure 1). Baseline characteristics were reported and analyzed based on data from the first enrollment for each patient. Table 1 outlines participant characteristics and demonstrates differences in characteristics between patients at the inpatient and outpatient sites.

**Table 1:** Differences in characteristics of study participants in hospital versus clinic patients

Characteristic	Hospital vs. clinic			*P* value
	Hospital, *n* (%)	Clinic, *n* (%)	Total, *n* (%)	
Age (months)				
0–6	9 (23.7)	28 (19.6)	37 (20.4)	0.884
7–12	5 (13.2)	28 (19.6)	33 (18.2)	
12–18	7 (18.4)	20 (14.0)	27 (14.9)	
19–24	7 (18.4)	22 (15.4)	29 (16.0)	
25–36	7 (18.4)	26 (18.2)	33 (18.2)	
37–48	2 (5.3)	10 (7.0)	12 (6.6)	
49–60	1 (2.6)	9 (6.3)	10 (5.5)	
Sex				
Male	15 (39.5)	81 (56.6)	96 (53.0)	0.059
Female	23 (60.5)	62 (43.4)	85 (47.0)	
Rotavirus vaccination				
No	14 (36.8)	17 (11.9)	31 (17.1)	< 0.001
Yes	24 (63.2)	126 (88.1)	150 (82.9)	
Rotavirus vaccine				
None	14 (36.8)	17 (11.9)	31 (17.1)	< 0.001
1 Dose	9 (23.7)	19 (13.3)	28 (15.5)	
2 Dose	15 (39.5)	107 (74.8)	122 (67.4)	
Ever breastfed*				
No	2 (5.3)	20 (14.0)	22 (12.2)	0.144
Yes	36 (94.7)	123 (86.0)	159 (87.8)	
Weight for height†				
Wasting	1 (2.7)	6 (4.2)	7 (3.9)	0.889
Normal	34 (91.9)	127 (89.4)	161 (89.9)	
Overweight	2 (5.4)	9 (6.3)	11 (6.2)	
Height for age‡				
Stunting	4 (10.8)	5 (3.5)	9 (5.0)	0.071
Normal	33 (89.2)	137 (96.5)	170 (95.0)	
Mother’s education level				
None	2 (5.3)	3 (2.1)	5 (2.8)	0.771
Primary	8 (21.1)	32 (22.4)	40 (22.1)	
Secondary	22 (57.9)	85 (59.4)	107 (59.1)	
University	6 (15.8)	23 (16.1)	29 (16.0)	

Pearson’s χ^2^ test was used to assess the association between age and recruitment location (hospital vs. clinic setting).

* Median (interquartile range) duration of breastfeeding was 5 months (3–6 months).

† Weight-for-height is defined based on WHO Child Growth Standards (WHO, 2008): wasting = weight-for-height <–2 SD; overweight = weight-for-height >+2.

‡ Height-for-age is defined based on WHO Child Growth Standards (WHO, 2008)^28^: stunting = height-for-age <–2 SD.

From the 181 patients, 28 (16.5%) were inpatients, and 142 (83.5%) were outpatients. The median participant age was 17 months (interquartile range: 8–26). A slight majority (53%) were male. Wasting was present in seven (3.9%) children, and nine (5%) were stunted. Eleven (6.1%) were overweight. Of the children with feeding data available, 159 (87.8%) had been breastfed for at least 1 month.

Among the children from the hospital, 14 (36.8%) did not have rotavirus vaccination compared with 24 (63.2%) who were vaccinated. In the clinic, 17 (11.9%) were unvaccinated and 126 (88.1%) were vaccinated. There was a statistically significant (Pearson χ^2^ = 16.6, *P* < 0.001) difference in vaccination rates between children at the hospital versus the clinic. In addition to the 14 (36.8%) hospitalized children who were completely unvaccinated for rotavirus, nine (23.7%) had received only one dose of the vaccine. Among patients from the clinic, in addition to the 17 (11.9%) who were completely unvaccinated for rotavirus, 19 (13.3%) had only one dose of the vaccine.

#### Rotavirus and enteropathogen detection from stool samples

There were 170 stool specimens available for analysis. Of these, 150 stool samples were from the first enrollment of the patients. Thirteen patients had more than one enrollment and therefore had more than one stool samples; seven patients had two stool samples, five had three samples, and one had four samples (Figure 1).

Among pathogens tested, rotavirus was detected most frequently with nine (32.1%) cases among hospitalized children and 16 (11.3%) among children from the NPS clinic. The prevalence of rotavirus was significantly higher among hospitalized children (Pearson χ^2^ = 8.1, *P* = 0.004). Rates of detection of rotavirus, *E. histolytica*, *Ascaris lumbricoides*, *Giardia lamblia*, and *Cryptosporidium* species are outlined in Table 2. Among patients with positive rotavirus results, three (50%) and 12 (80%) patients from the hospital and NPS clinic, respectively, were fully vaccinated.

**Table 2:** Frequency of detection of *Ascaris lumbricoides*, *Giardia lamblia*, *Entamoeba histolytica*, rotavirus, and *Cryptosporidium parvum*

Pathogen	Hospital, *n* (%)	Clinic, *n* (%)	Total, *n* (%)	*P* value
*Ascaris lumbricoides*				
Negative	28 (100.0)	139 (97.9)	167 (98.2)	0.438
Positive	0 (0.0)	3 (2.1)	3 (1.8)	
*Giardia lamblia*				
Both negative	26 (92.9)	127 (89.4)	153 (90.0)	0.526
Stool microscopy only	0 (0.0)	2 (1.4)	2 (1.2)	
Both positive	2 (7.1)	6 (4.2)	8 (4.7)	
CerTest rapid test only	0 (0.0)	7 (4.9)	7 (4.1)	
*Entamoeba histolytica*				
Both negative	20 (71.4)	107 (75.4)	127 (74.7)	0.698
Stool microscopy only	8 (28.6)	33 (23.2)	41 (24.1)	
CerTest rapid test only	0 (0.0)	2 (1.4)	2 (1.2)	
Rotavirus				
Negative	19 (67.9)	126 (88.7)	145 (85.3)	0.004
Positive	9 (32.1)	16 (11.3)	25 (14.7)	
*Cryptosporidium parvum*				
Negative	28 (100.0)	134 (94.4)	162 (95.3)	0.198
Positive	0 (0.0)	8 (5.6)	8 (4.7)	

Pearson’s χ^2^ test was used to assess the association between pathogen and recruitment location (hospital vs. clinic setting). CerTest rapid test was used for detection of rotavirus, *C. parvum*, *G. lamblia*, and *E. histolytica*; *G. lamblia* and *E. histolytica* were also detected using stool microscopy; and *A. lumbricoides* was detected using stool microscopy.

### Polymerase chain reaction results and seasonality of rotavirus.

Among 70 stool samples sent for PCR testing (seven from hospitalized patients and 63 from outpatients), one or more pathogens was detected in 62 (88.6%). Of those, only seven (11.3%) had a single pathogen detected. Pathogenic *E. coli* species were the most commonly detected organisms (see Table 3).

**Table 3:** Frequency of pathogen identification by polymerase chain reaction (*N* = 70)

Pathogen name	Single pathogen		Multiple pathogen		Total
	Clinic	Hospital	Clinic	Hospital	
*Campylobacter* (*C. jejuni/C. coli/C. upsaliensis*)	0	1	0	12	13
*Clostridioides* (*Clostridium difficile* [*toxin A/B*])	0	0	0	5	5
*Plesiomonas shigelloides*	0	0	1	3	4
*Salmonella*	0	0	1	3	4
*Yersinia enterocolitica*	0	0	0	1	1
*Vibrio* (*V. parahaemolyticus/V. vulnificus/V. cholerae*)	0	0	0	7	7
*Vibrio cholera*	0	0	0	6	6
*Enteroaggregative Escherichia coli*	0	1	4	31	36
*Enteropathogenic E. coli*	0	2	2	30	34
*Enterotoxigenic E. coli lt/st*	0	0	2	18	20
*Shiga-like toxin-producing E. coli stx1/stx2 E. coli O157*	0	0	4	7	11
*Shigella/enteroinvasive E. coli*	0	0	5	24	29
*Cryptosporidium*	0	2	1	7	10
*Cyclospora cayetanensis*	0	0	0	0	0
*Entamoeba histolytica*	0	0	0	0	0
*Giardia lamblia*	0	0	0	11	11
Adenovirus F40/41	0	0	0	2	2
Astrovirus	0	0	1	2	3
Norovirus GI/GII	0	1	1	11	13
Rotavirus A	0	0	0	2	2
Sapovirus (I, II, IV, and V)	0	0	0	18	18

No pathogens were detected in eight of the 70 samples assessed. Only seven samples had single pathogen detection. *Cyclospora cayetanesis* and *Entamoeba histolytica* were not detected.

Discordance between PCR test results and standard laboratory evaluations was common. *Giardia lamblia* was detected in 11 (15.7%) PCR samples; three and two of those sample were also positive for *G. lamblia* using stool microscopy and CerTest rapid test, respectively. Between stool microscopy and CerTest rapid test, there was one additional *G. lamblia* detection in the stool microscopy test sample. *Cryptosporidium* species was in 14 (20%) of the PCR samples, but only one of those also showed *Cryptosporidium* species on the CerTest rapid test. Four (5.7%) of detected *Cryptosporidium* species was detected only by the CerTest rapid test. Rotavirus was detected in two (2.9%) of the PCRs. Both stool samples that tested positive for rotavirus by PCR also tested positive on CerTest rapid test. However, there was one additional sample that tested positive on CerTest rapid test that was not detected by the PCR. *E. histolytica* was not detected in any PCR samples despite being reported in 23 (32.9%) of the stool microscopy samples and one (1.43%) CerTest rapid test.

**Figure 2.**
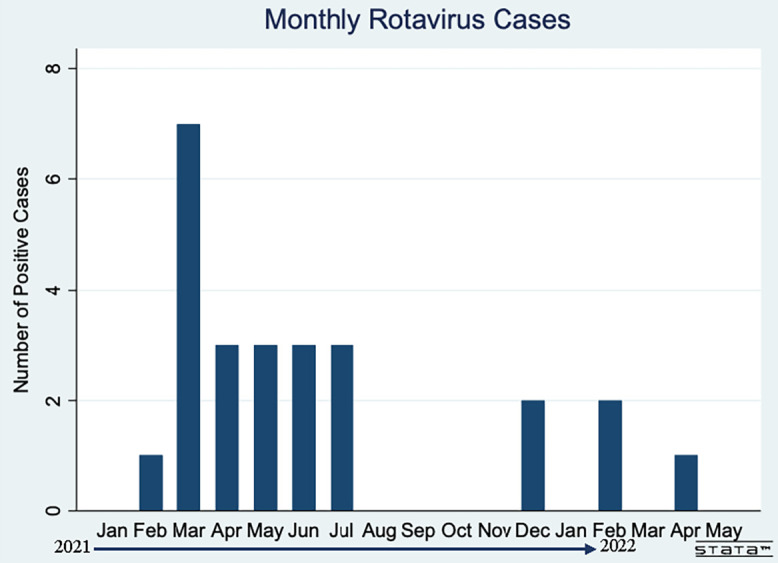
Monthly positive rotavirus cases among all participants. March 2021 has the highest number of positive cases (seven of 25). Three cases were detected each month between April and July. There were two positive cases each for December 2021 and February 2022. There was one positive case each for February 2021 and April 2022.

Of the 25 rotavirus samples detected over the course of the study, seven (28%) were detected in March 2021. Three cases were detected each month between April and July, with the additional cases scattered throughout the rest of the year (Figure 2).

## DISCUSSION

Despite the availability of rotavirus vaccination, rotavirus remained a common cause of acute gastrointestinal illness among children under 5 years of age in our cohort in Consuelo, Dominican Republic. Children who were not fully vaccinated against rotavirus were more likely to be hospitalized for acute gastroenteritis and were more likely to test positive for rotavirus than their fully vaccinated peers with acute gastroenteritis. The differences in rotavirus vaccination rates between inpatients and outpatients in this study may be partly attributed to the NPS clinic’s immunization outreach. The vaccination rate among the NPS cohort has gone up from 49% in 2009 to > 95% in 2015 for all recommended vaccines for children under 5 years of age.^29^ Because Hospital Municipal Dr. Angel Ponce Pinedo serves a larger catchment area, undervaccinated subpopulations, such as the children of migrant workers living in nearby batey communities, would have been included in our inpatient but not our outpatient population. Many batey populations lack potable water, electricity, and sanitation facilities and have reduced access to vaccination and basic health care.^30^

Rotavirus was detected more frequently among patients from the hospital, which likely was related to both the higher vaccination rate in the NPS population and the fact that rotavirus infection results in more severe symptoms in unvaccinated children.^31^ Rotavirus was detected in 14.7% of all the patients and in 32.1% of all hospitalized patients in the study. Rotavirus vaccine has been shown to decrease serious adverse events related to gastroenteritis, such as diarrhea, vomiting, dehydration, hypovolemic shock, and hospital admission.^31^ In this sample, 74.8% of the clinic patients and 39.5% hospitalized patients had received the recommended two doses of the vaccine. As expected, a lower proportion of hospitalized patients compared with clinic patients had received rotavirus vaccination. In the Dominican Republic, the most commonly detected rotavirus genotypes include G1P[8] and G2P[4]. However, G3P[8], G1P[6], and G3P[6] genotypes and mixed infections have also been detected at lower frequencies.^11^ The live attenuated vaccine efficacy has been reported to be 91% against G1P[8] rotaviruses, 67% against G2P[4], indicating that the vaccine protects against the most commonly reported genotypes in the Carribean.^2^

Overall, there was a decrease in rotavirus detection rate compared with existing data before rotavirus vaccine was introduced. The proportion of acute gastroenteritis hospitalizations caused by rotavirus was estimated to be 62% before the vaccine introduction,^12^ which is significantly higher than our findings of 32.1% in the hospital setting in Consuelo, Dominican Republic. However, our findings are slightly higher than recent global trends where rotavirus related hospitalization among children under 5 years was 20% 4 years after rotavirus vaccine introduction.^32^ One possible reason for our findings is that in countries with high child mortality rates, vaccine efficacy may be lower and may decrease faster over time.^33^ Overall immunization rates might also differ drastically by region. In our study, 39.5% of children from the hospital and 74.8% of children from the clinic had received the recommended two doses of the vaccine. However, the WHO’s estimation of rotavirus vaccination rate in 2021 in the Dominican Republic was 80%.^19^ These differences might be explained by lack of available data for vaccine estimation by region. For example, in Peru, after the introduction of rotavirus vaccine, acute gastroenteritis mortality and morbidity varied by region; some regions also had fewer data available.^34^ This suggests that it is important to consider the location and population served by each institution to contextualize our findings. The hospital serves a diverse population from various regions, whereas the NPS clinic serves 11 specific neighborhoods that receive support from the clinic.

The PCR testing on a subset of the stool samples suggested that the sensitivity of commonly available laboratory tests was suboptimal to detect many pathogens, particularly bacterial pathogens. Overall, PCR results showed higher detection of pathogens than the CerTest rapid test and stool microscopy. In a study by Cybulski et al., FilmArray led to increased detection of *E. histolytica* and *G. lamblia* when compared with ova and parasites examination of the same specimen.^35^ In our study, *G. lamblia*, *Cryptosporidium* species, and rotavirus A were detected at a higher rate by PCR than by the laboratory analyses that are more commonly available in this setting. Although PCR is more sensitive and can detect a broad range of pathogens, the higher rate of detection could also be due to asymptomatic fecal shedding of nucleic acids instead of an active infection.^36^ In our study, *Entamoeba* species were detected mostly by stool microscopy. *Entamoeba histolytica* was detected in 0% of PCR results and 1.4% by CerTest rapid tests. *Entamoeba* overall were found in 32.9% of microscopy samples, however, likely reflecting the presence of the nonpathogenic *Entamoeba dispar*, which is not distinguishable from *E. histolytica* by direct microscopic examination.^37^

We did not find any clear rotavirus seasonality. We observed a small spike in rotavirus cases during March 2021; however, there were no cases detected during March 2022. In Haiti, Desormeaux et al. observed a clear annual rotavirus seasonality, with the greatest activity in December through April.^12^ In Latin American countries, changes in rotavirus seasonality differ by country, but the highest number of infections are observed during colder and drier times of the year.^38^ In the Dominican Republic, the drier and slightly cooler season is between December through April.

A study conducted in El Salvador found that one dose of vaccine confers ∼50% protection against rotavirus related hospital admission.^5^ This was consistent with our findings that a smaller proportion of those hospitalized with rotavirus were fully vaccinated, compared with outpatients with rotavirus.

Our analysis has several limitations. First, our findings from the NPS clinic may not be generalizable to other outpatient settings in the Dominican Republic, given that the NPS clinic puts significant resources into maximizing vaccination rates among children in the 11 neighborhoods in its catchment area. Second, we assessed rotavirus seasonality based only on a single year of data. Third, because the PCR-positive results were not confirmed by independent methods to discriminate active infection from asymptomatic, we cannot accurately distinguish between fecal shedding of nucleic acids and active cases. The results do, however, suggest that stool culture yield may be suboptimal in this setting.

Given the higher percentage of rotavirus detected among patients from the hospital and the relatively low vaccination rate among those patients, continued efforts to optimize vaccination are necessary to reduce moderate and severe rotavirus disease in this population. Future studies should look at strategies to improve rotavirus vaccination coverage among children in the most marginalized communities in the Dominican Republic.
